# Spermidine exogenous application mollifies reproductive stage heat stress ramifications in rice

**DOI:** 10.3389/fpls.2022.1027662

**Published:** 2022-12-02

**Authors:** Sourabh Karwa, Jyoti Taunk, Sadhana Maurya, Adhip Das, G. K. Krishna, Sunder Singh Arya, Awadhesh Kumar, Sudhir Kumar, Pramod Kumar, Viswanathan Chinnusamy, Madan Pal

**Affiliations:** ^1^ Division of Plant Physiology, ICAR-Indian Agricultural Research Institute, New Delhi, India; ^2^ Department of Botany, Maharshi Dayanand University, Rohtak, Haryana, India; ^3^ Department of Biotechnology, University Centre for Research and Development (UCRD), Chandigarh University, Mohali, Punjab, India; ^4^ Department of Plant Physiology, College of Agriculture, Kerala Agricultural University, Thrissur, India; ^5^ Crop Physiology and Biotechnology Division, Indian Council of Agricultural Research-National Rice Research Institute (ICAR-NRRI), Cuttack, Odisha, India

**Keywords:** Antioxidant enzymes, flowering, heat stress, polyamine, rice, spermidine, spikelet fertility

## Abstract

**Introduction:**

Rice productivity is severely hampered by heat stress (HS) which induces oxidative stress in this crop. This oxidative stress can be alleviated using various exogenous chemicals, including spermidine (Spd). Therefore, the present study was carried out to characterize HS components and to elucidate the role of exogenous Spd application in rice at the flowering stage.

**Methods:**

Two contrasting rice genotypes, i.e. Nagina22 (N22) and Pusa Basmati-1121 (PB-1121) were placed in temperature tunnels and exposed to HS (38–43°C) with and without Spd (1.5 mM) foliar application during the heading stage till the end of the anthesis stage.

**Result:**

Heat stress induced the production of H_2_O_2_ and thiobarbituric acid reactive substances, which resulted in lower photosynthesis, spikelet sterility, and reduced grain yield. Interestingly, foliar application of Spd induced antioxidant enzyme activities and thus increased total antioxidant capacity resulting in higher photosynthesis, spikelet fertility, and improved grain yield under HS in both genotypes. Under HS with Spd, higher sugar content was recorded as compared to HS alone, which maintained the osmotic equilibrium in leaf and spikelets. Spd application initiated *in vivo* polyamine biosynthesis, which increased endogenous polyamine levels.

**Discussion:**

This study corroborates that the exogenous application of Spd is promising in induction of antioxidant defence and ameliorating HS tolerance in rice *via* improved photosynthesis and transpiration. Thereby, the study proposes the potential application of Spd to reduce HS in rice under current global warming scenario.

## Introduction

Rice is the most important staple food crop cultivated over 167.13 mha across the globe (http://www.fao.org/faostat/en/#compare, accessed on 20^th^ January 2021), feeding over 3.5 billion people (FAO, 2014). Rice production is facing an unprecedented challenge with increasing global mean temperature and diminishing freshwater resources. The climate change scenario predicted a rise in temperature by 1.6-4.4°C by 2100 and a more frequent occurrence of heat episodes during the sensitive growth stages such as flowering in case of rice (Teixeira et al., 2013; IPCC, 2021). At this stage, heat stress (HS) could result in anther dehiscence, poor pollen germination, ultimately reducing spikelet fertility and grain yield (Hu et al., 2021). Under HS, the plant follows various tolerance and avoidance mechanisms to cope with the HS conditions (Hasanuzzaman et al., 2013), including high transpirational cooling (Karwa et al., 2020).

Heat stress induces oxidative stress at the tissue level by overproducing reactive oxygen species (ROS), damaging protein function and membrane integrity (Zhao et al., 2018). In addition, gas exchange, membrane permeability, sugar and starch accumulation are also vulnerable to HS at the flowering stage in rice and other crops (Paupière et al., 2014). Under stress conditions (HS), the plant responds *via* an orchestrated and complex network of different plant regulators that plays a vital role in acquiring tolerance (Garay-Arroyo et al., 2012). Growth regulators or stress hormones such as salicylic acid, proline, betaine and brassinosteroids can ameliorate the negative effect of oxidative stress by fortifying antioxidant defence machinery (Ghosh et al., 2022; Hossain et al., 2022; Raza et al., 2022). Recently, a new class of small and low molecular weight aliphatic amines, i.e. polyamines, have been suggested to improve plant tolerance towards various abiotic stresses such as drought, salinity and heavy metals (Capell et al., 2004; Zhao et al., 2004; Handa and Mattoo, 2010; Do et al., 2014; Soudek et al., 2016). There are three common and naturally occurring polyamines, namely putrescine (Put), spermidine (Spd) and spermine (Spm). Spd has been documented to directly act as a stress protecting compound (Chen et al., 2021) and is involved in various stress signal transduction pathways (Kasukabe et al., 2004). The potential of Spd to improve plant tolerance towards various abiotic stresses like drought (Kubiś, 2001; Kubiś, 2003), salinity (Roy et al., 2005; ElSayed et al., 2018), heat (Tian et al., 2009) and submergence (Liu et al., 2015) is well documented. Spd application on rice seedlings under HS has reduced H_2_O_2_, proline and malondialdehyde (MDA) contents (Mostofa et al., 2014). Spd alleviated drought stress in maize seedlings by protecting the photosynthetic apparatus, which improved their photosynthetic performance (Li et al., 2018). However, the effect of Spd in ameliorating HS has not been investigated in rice, particularly at the flowering stage. Therefore, the present study was aimed to analyse the impact of HS on rice growth and yield metrics; and to authenticate the hypothesis that exogenous Spd application induces HS tolerance in rice at the reproductive stage.

## Materials and methods

### Plant material and growth conditions

Effects of HS on physio-biochemical traits and mitigation response of Spd against HS were studied in two rice genotypes *viz.* Nagina22 (N22) (03911) and Pusa Basmati-1121 (PB-1121) (**Table 1**). The experiments were conducted in the Pot culture facility, Division of Plant Physiology, Indian Council of Agricultural Research (ICAR)-Indian Agricultural Research Institute (IARI), New Delhi, during *Kharif*, 2016. Plants were raised in pots (14 cm diameter and 12 cm height) by transplanting 21 days old rice seedlings from the nursery. Each pot was filled with 20 kg of clay-loam soil supplemented with farmyard manure (800 g pot^-1^). The N: P: K fertilizer was provided as (NH_4_)_2_SO_4_ (0.375 g kg^-1^), SSP (0.075 g kg^-1^) and KCl (0.075 g kg^-1^), respectively, in split doses. Another dose of N (0.125 g kg^-1^ soil) was top-dressed 25–30 d. Fifteen biological replicates were followed in all the experiments. All pots were arranged randomly as per the layout obtained through Statistical Tool for Agricultural Research (STAR; Version: 2.0.1) and placed at the Net house facility. Plants were kept under flooded conditions (water 3-5 cm above the soil surface) till their physiological maturity. Heat stress treatment was commenced at the heading stage by shifting the pots in a temperature tunnel, with an average temperature of ~5˚C more than the ambient temperature and was kept for at least ten days, covering flowering and post-flowering representative genotypes. Sampling was done on the main tiller, tagged before stress treatments. No significant insects or pests were observed during the experiment. For the present study, three sets of treatments were designed as (1) C: Ambient temperature (Control); (2) HS: Heat stress (elevated temperature) and (3) HS+Spd: Heat stress (elevated temperature) with Spd (1.5mM) application.

**Table 1:** Rice genotypes tested under this study.

Genotype	Origin	Species	Stress tolerance	Reference(s)
Nagina22 (N22) (03911)	India	*Oryza sativa* aus	Heat tolerant	Prasad et al. (2006); Jagadish et al. (2008); Jagadish et al. (2010), Selote and Khanna-Chopra (2004)
Pusa Basmati-1121 (PB-1121)	India	*Oryza sativa* indica	Heat sensitive	Singh et al. (2018)

### Heat stress treatment in the high-temperature tunnel

The plants were grown in ambient conditions until booting and were shifted to high temperature tunnel (HTT) at the heading stage. The design and control system of the temperature tunnel was followed as described by Sinclair et al. (1995). The air temperature and relative humidity (RH) were recorded on a real-time basis at every 30 min interval by MINCER (Micrometeorological Instrument for the Near-Canopy Environment of Rice) as per Fukuoka et al. (2012). MINCER was installed at the centre of the HTT, and sensor height was kept at the height of the rice canopy. For comparison, data represented in the figures includes the HS period (heading to 100% flowering) for both ambient net house and HTT. The ambient and HTT was recorded diurnally and expressed as the mean day (0700–1800 h)/night (1800–0700 h) temperature for both the experiments.

### Growth environments in ambient and HTT

The mean maximum temperature (T_max_) was 34.3°C (SD ± 1.68), and mean minimum temperature (T_min_) was 22.6°C (SD ± 0.78) in ambient conditions, while in HTT, T_max_ was 39.2°C (SD ± 2.78), and T_min_ was 26.3°C (SD ± 1.03) (**Supplementary Figures 1A, B**). There was an increment of 4.8°C during day and 3.7°C at night over ambient conditions in HTT. In the present study, both the genotypes differed in the onset of flowering, first observed in N22, followed by PB-1121.

### Exogenous Spd application

Spd (Himedia, India) stock solution (1.5 mM) was prepared in distilled water containing 0.01% (v/v) Tween-20 as surfactant to enhance foliar adhesion. For foliar application, 20–25 ml of solution per plant was sprayed during 1700 to 1900 h before HS.

### Sampling

The flag leaf and spikelets were used as samples for evaluating all the biochemical parameters, and the evaluations were performed at 100% flowering of the respective genotype. Only the tagged panicles were harvested to avoid sampling error. The samples were flash-frozen in liquid nitrogen and stored at -80°C until further analysis.

### Evaluation of spikelet fertility and yield-associated traits

Data on spikelet fertility, grain weight plant^-1^ and 1000 grain weight were recorded according to Prasad et al. (2006). At physiological maturity, panicle and biomass were separately harvested and packed into separate envelopes. Panicle was sun dried for 3-4 days or till the constant weight was achieved. Then grains were threshed from the panicles and weight was measured using analytical balance (model: BSA124S-CW, Sartorius AG, Germany) which was expressed as grain weight plant^-1^. From the harvested panicles, five random panicles per plants were selected and counting of filled and unfilled grains per panicle was performed manually. The ratio of filled grains with total number of spikelet per panicle was expressed in percentage. The 1000 grain weight of seeds was counted manually from final thrashed grains and weight was calculated using analytical balance.

### Physiological parameters

#### Measurement of relative water content and membrane stability index

To derive RWC, the fresh weight (FW) of the flag leaf was recorded, then it was hydrated overnight to take the turgid weight (TW) and oven-dried for two days for dry weight (DW) analysis. RWC was calculated according to Barrs and Weatherley (1962).

Fresh leaf samples were cut into pieces of equal sizes and transferred to test tubes containing distilled water to obtain MSI. The samples were incubated in the water bath at 45°C for 60 min. After cooling, the conductivity (C1) of the solution was measured. Samples were further kept at 100°C for 10 min, and then conductivity was again measured after cooling. After that, MSI was calculated according to Sairam et al. (2002).

#### Measurement of net photosynthesis rate and associated gas exchange parameters

Gas exchange parameters such as net photosynthesis rate (*P_N_*; μmolCO_2_ m^−2^s^−1^), stomatal conductance (*gS*; molH_2_Om^−2^s^−1^) and transpiration rate (*E*; mmolH_2_Om^-2^s^-1^) were measured using the LI-COR portable photosynthesis system (LI-6400 model, LI-COR, Lincoln, NE). The gas exchange measurements were recorded from the flag leaf between 0830 and 1130 hours. The CO_2_ concentration of the reference air entering the leaf chamber (3 x 2 cm; Model 6400–02B; LI-COR Inc. USA) was adjusted with a CO_2_ mixer control unit, keeping it at 400 µmol mol^–1^ with a constant flow rate of 500 µmol s^–1^. Measurements were recorded with a Photosynthetic photon flux density (PPFD) at 1200 mmol m^–2^s^–1^ supplied with red LEDs (LI-6400–02; LI-COR Inc.). Chamber block temperature was set as per ambient conditions, and the RH was kept close to 60% (Bahuguna et al., 2018).

### Biochemical parameters

#### Measurement of sugar and starch content

Sugar and starch contents were measured in flag leaf and spikelets spectrophotometrically, as described by McCready et al. (1950). In brief, samples were grounded in pestle motar using liquid nitrogen. Sample (0.1g) was then mixed with 80% ethanol (V/V) and centrifuged. The extract was collected and sugar analysis was performed using anthrone reagent. Remaning pellet was dried after sugar anlaysis and perchloric acid was added for starch extraction from the sample. Again it was centrifuged and further analyzed using anthrone reagent. Quantitative estimation of these samples was performed using UV-visible spectrophotometer at an absorbance of 630 nm (Model: Specord Bio. 200, AnalytikJena, Germany).

#### Measurement of hydrogen peroxide and thiobarbituric acid reactive substances

The H_2_O_2_ and TBARS contents were calculated in both flag leaves and spikelets. H_2_O_2_ content (μmolg^−1^FW) was measured spectrophotometrically as described by Alexieva et al. (2001). The concentration of H_2_O_2_ was calculated from the standard curve plotted against Hydrogen peroxide solution (Merck, Germany). TBARS content (μmolg^−1^FW) was measured spectrophotometrically as described by Larkindale and Knight (2002) and calculated using an extinction coefficient of 155 mM cm^−1^.

#### Measurement of total antioxidant capacity

TAC was measured by Ferric reducing antioxidant power (FRAP) assay as per Benzie and Strain (1999) and expressed as the ferric-reducing ability of mmol L^-1^ FeSO_4_. In brief, sample was grounded using liquid nitrogen in pestle and mortar. Grounded sample was aliquoted with 70% ethanol (v/v). After centrifugation, supernatant was collected and further analyzed using FRAP reagent (3 ml) in dark. It was then incubated in water bath for 10 min at 37^0^C and absorbance was recorded at 593 nm using UV-visible spectrophotometer (Model: Specord Bio. 200, AnalytikJena, Germany).

### Measurement of antioxidant enzyme activity

#### Total soluble protein extraction

Total protein was extracted from flag leaf and spikelets to estimate antioxidant enzyme activities. The samples were homogenized and transferred to microcentrifuge tubes containing ice-cold potassium phosphate buffer (0.1 M, pH=7.0) and 0.1 mM disodium ethylene diamine tetra acetate dehydrate (Na-EDTA). In case of protein extraction for estimation of ascorbate peroxidase activity, Na-EDTA was replaced by 10 mM ascorbate in the buffer. The homogenate was then centrifuged at 18,400 g for 20 min at 4°C, and the supernatant was used as crude enzyme extract. Protein content was estimated following the Bradford method (Bradford, 1976).

#### Antioxidant enzyme assays

Superoxide dismutase (SOD; EC 1.15.1.1) activity was assayed by monitoring the inhibition of photochemical reduction of nitro blue tetrazolium (NBT) following the method of Jiang and Zhang (2002). It was expressed as one unit of SOD activity mg^−1^ protein. Catalase (CAT; EC 1.11.1.6) activity was assayed by measuring the disappearance of H_2_O_2_ at 240 nm (extinction coefficient = 39.4 mM^−1^cm^−1^) and was expressed as μmol of H_2_O_2_ consumed mg^−1^ protein min^−1^ (Jiang and Zhang, 2002). Ascorbate peroxidase (APX; EC 1.11.1.11) activity was determined by decrement in absorbance at 290 nm, as described by Sharma and Dubey (2004). The enzyme was quantified using the extinction coefficient of 2.8 mM^−1^cm^−1^ and expressed as μmol ascorbate mg^−1^ protein min^−1^. Guaiacol peroxidase (GPX; EC 1.11.1.7) activity was determined as described by de Azevedo Neto et al. (2006). Enzyme activity was quantified using molar extinction coefficient (26.6 mM^−1^cm^−1^) to calculate the formation of tetraguaiacol and was expressed as μmol H_2_O_2_ mg^−1^ protein min^−1^.

### Estimation of endogenous free-polyamines

The extraction and estimation of free polyamines (Put, Spd and Spm) were performed following the perchloric acid method given by Flores and Galston (1982). Derivatization of free polyamines was done by alkali (NaOH) treatment followed by benzoylation. The reaction was terminated using saturated NaCl. Cold diethyl ether (2 mL) was used for extracting polyamines from benzyl polyamine. The ether phase was collected and evaporated to dryness and re-dissolved in 100 µL High-Performance Liquid Chromatography (HPLC) grade methanol (Merck, Germany). HPLC analysis was performed using the reverse-phase (C18) column (Agilent) on Agilent 1100. Benzoylated polyamine sample (20 µL) was injected by an autosampler with a flow rate of 1 mL/min of mobile phase (acetonitrile: water; 52:48 v/v) (Merck, Germany). The area and data retrieval calculations were performed using CHEM STATION for LC system Rev B.040.3 (16) software. The concentrations of individual polyamines were calculated from the standard curve plotted against HPLC grade standards of Put, Spd and Spm (Sigma chemicals, USA) and expressed as nmol g^−1^ FW (**Supplementary Figure 2**).

### Statistical analysis

Data were analyzed by two-way Analysis of variance (ANOVA) in a completely randomized design using Statistical Package for the Social Sciences (SPSS) 13.0 (LEAD Technologies Inc.) to compare the differences between cultivars, treatments and their interaction. *Post-hoc* test (Tukey’s) was performed to retrieve the difference between treatments and genotypes at least significant (LSD) of 5% and 1%.

## Results

### Effect of Spd on yield-related parameters under HS

The reduction was recorded in spikelet fertility, grain yield plant^-1^ and 1000 grain weight under HS. Spikelet fertility and grain yield plant^-1^ showed significant genotype (G) x treatment (T) interaction (P<0.05-0.001) under HS at the flowering stage (**Table 2**). Heat stress significantly reduced spikelet fertility in both the genotypes, as 23.5% and 6.5% reduction was recorded in PB-1121 and N22, respectively with respect to the control. Application of Spd significantly decreased yield penalty under HS. Compared to HS alone (without any exogenous application of polyamine), Spd application under HS significantly improved spikelet fertility by 9.5% and 2.0% in PB-1121 and N22, respectively. Under HS, maximum reduction of 67.3% grain yield plant^-1^ was recorded in case of PB-1121, and minimum reduction (8.1%) was recorded in case of N22 (**Table 2**). Under HS, a significant reduction in 1000 grain weight was recorded in PB-1121 (28.8%) while a lower reduction was observed in N22 (8.1%) as compared to the ambient conditions. In addition, 1000 grain weight showed significant (P<0.001) treatment interaction (**Table 2**). Spd application under HS resulted in comparatively lesser reductions of 1000 grain weight which was estimated to be 11% and 6.5% in PB-1121 and N22 genotypes, respectively.

**Table 2:** Yield components (spikelet fertility, grain yield plant^-1^ and 1000 grain weight) of rice genotypes under heat stress.

Genotype	Treatment	Spd application	Grain yield plant^-1^ (g)	Spikelet fertility (%)	1000 grain weight (g)
N22	Ambient	-Spd	51.2 ± 3.66	93.7 ± 1.26	20.70 ± 0.12
	HS	-Spd	47.1 ± 4.92	87.6 ± 1.48	19.02 ± 0.59
		+Spd	48.8 ± 4.43	89.3 ± 0.97	20.27 ± 0.37
PB-1121	Ambient	-Spd	55.8 ± 4.19	89.8 ± 0.59	22.97 ± 0.92
	HS	-Spd	18.2 ± 2.01	68.7 ± 2.56	16.37 ± 0.37
		+Spd	29.0 ± 5.83	75.2 ± 3.99	17.72 ± 0.09
Lsd<0.05		G	7.71***	2.32***	0.89*
		T	8.95***	2.81***	1.09***
		G X T	12.66**	3.98**	1.504***

Each point represents the mean of five replicates with their ± SE. Least significant difference (Lsd) P<0.05, 0.01 and 0.001 were denoted by *, **, and*** respectively. G, Genotypes; T, Treatment; HS, Heat stress; SE, Standard error; Spd, Spermidine.

### Effect of Spd on net photosynthesis rate and associated gas exchange parameters

**Figure 1:**
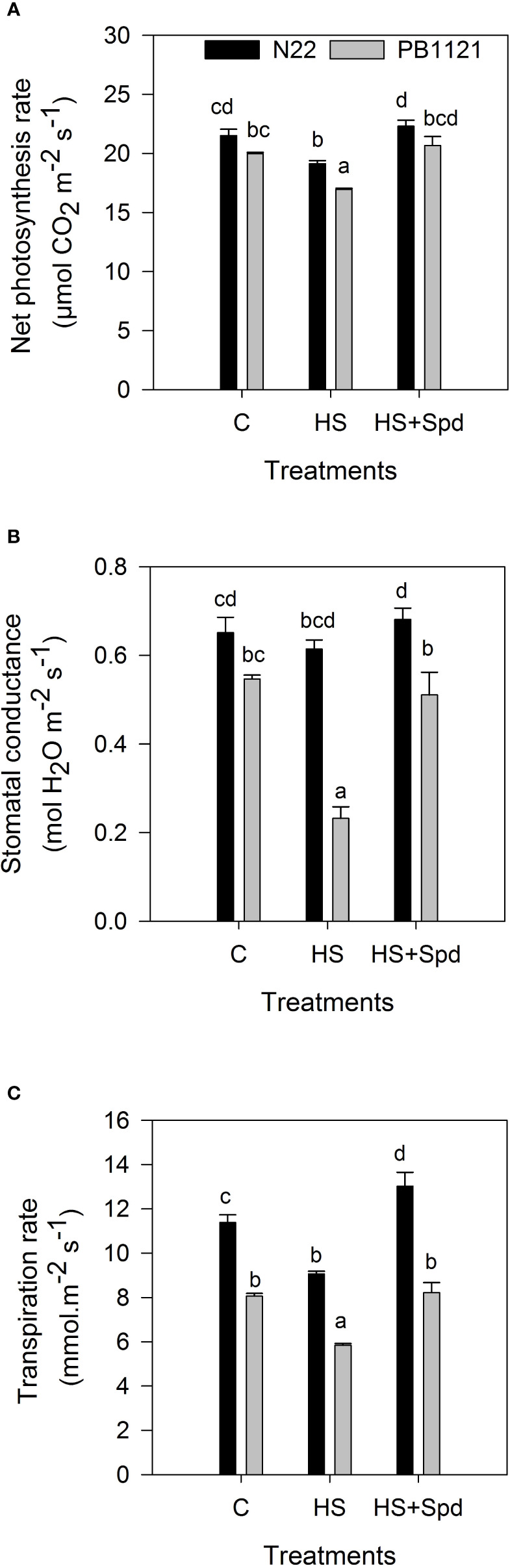
Net Photosynthesis rate **(A)**, Stomatal conductance **(B)** and Transpiration rate **(C)** under different treatments in the contrasting rice genotypes (N22 and PB-1121). Bars indicate mean ± SE. Comparison of means was obtained from Tukey’s honestly significant difference test where means with the same letter are not significantly different at 5%. C, Control; HS, Heat stress; Spd, Spermidine. Least significant difference values at 5% for comparison are given in **Supplementary Table 1**.

*P_N_* varied significantly in G and T (P<0.001) (**Figure 1A**; **Supplementary Table 1**). Within the genotypes, *P_N_* reduced on the 7^th^ day of HS in the range 11–15% with respect to the C (**Figure 1A**). There was a significant reduction in *P_N_* by 15.1% in PB-1121 and by 11.1% in N22 under HS with respect to the ambient. Spd application significantly recovered the *P_N_* in tested genotypes. When compared with HS, the maximum rescue of *P_N_* was recorded in N22 (16.5%) followed by PB-1121 (21.9%) under HS+Spd. The *gS* showed significant variation within G x T interaction (P<0.001) (**Figure 1B**; **Supplementary Table 1**). Compared to the ambient, HS showed reductions in the range of 5.0-57%, with a minimum reduction in N22 and a maximum in PB-1121. Spd application significantly improved *gS* by 10.9% and 119.9% in N22 and PB-1121, respectively, compared to HS. A similar trend was recorded in E, with significant G x T interaction on the 7^th^ day of HS (**Figure 1C**; **Supplementary Table 1**). HS+Spd significantly increased E in both the tested genotypes. When compared with HS, a higher increase in E was recorded in N22 (43%) and lower in PB-1121 (41%).

### Effect of Spd on RWC and MSI

RWC showed significant variation between genotypes (G) and treatment (T) (P<0.001) (**Table 3**). Within the two genotypes, RWC reduction was recorded in the range of 18–30% under HS with respect to the ambient. Spd application significantly increased water retention efficiency under HS+Spd (9-19%) over corresponding HS treated plants on the 7^th^ day of heat exposure. Compared to HS alone, RWC increased by 19.0% in PB-1121 and 9.2% in N22 after Spd application under HS. MSI also showed a significant G and T (P<0.001) response (**Table 3**). Across the genotypes, reduction in MSI was recorded in a range of 16.0–24.0% under HS. Spd application significantly improved membrane stability by 6.0% and 5.0% in PB-1121 and N22, respectively.

### Effect of Spd on H_2_O_2_ and TBARS

To understand the impact of heat stress on flag leaf and spikelets and the protective role of Spd, H_2_O_2_ and TBARS were measured at the flowering stage. H_2_O_2_ accumulation showed significant G x T interaction (P<0.001) in flag leaf and spikelet (**Table 3**) with higher accumulation in former. The flag leaf of PB-1121 showed H_2_O_2_ accumulation of 15.2 µmol g^-1^ FW, while in case of spikelet, 11.7 µmol g^-1^ FW was observed under HS on the 7^th^ day of heat exposure. When compared with HS alone, Spd application reduced H_2_O_2_ production in both the genotypes. Higher H_2_O_2_ accumulation was recorded in flag leaf and spikelets of PB-1121 (13.7 and 10.8 µmol g^-1^FW, respectively) under HS+Spd as compared to N22 (12.4 and 10.5 µmol g^-1^FW, respectively). TBARS, which was calculated in terms of MDA accumulation, showed significant G x T interaction (P<0.001) in flag leaf and spikelet (**Table 3**). TBARS accumulated maximally in both flag leaf and spikelets with 44.3% and 118.7%, respectively, in PB-1121 under HS compared to ambient. Compared with HS, Spd application reduced MDA accumulation in N22 (10.0% and 0.6%) and PB-1121 (13% and 26%) in flag leaf and spikelets, respectively.

### Effect of Spd on sugar and starch content

**Figure 2:**
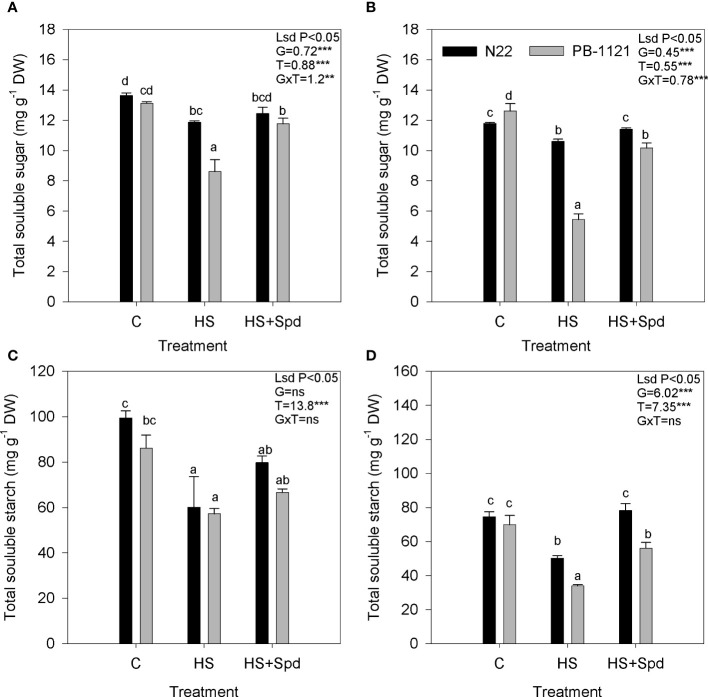
Effect of heat stress on sugar and starch contents under different treatments in rice flag leaf **(A, C)** and spikelets **(B, D)**, respectively. Each point represents mean of five replicates. C, Control; DW, Dry weight; HS, Heat stress; Spd, Spermidine. Significance level: **P < 0.01; ***P < 0.001; ns, nonsignificant.

Total soluble sugar content in flag leaf and spikelets showed significant variation (P<0.01-0.001) among genotypes and treatment (**Figure 2**) after exposure to HS. On the 7^th^ day of HS, significant sugar reduction was recorded in flag leaf (13% and 34%) and spikelets (10% and 57%) of N22 and PB-1121, respectively with respect to the control. Heat stress with Spd foliar application enhanced sugar accumulation in N22 (4% and 8%) and PB-1121 (36% and 86%) in flag leaf and spikelets, respectively with respect to HS. Similarly, starch content showed significant variation (P<0.05-0.001) among the genotypes and under treatment. On the 7^th^ day of HS, a significant reduction of starch content was recorded in flag leaf (39% and 34%) and spikelet (32.6% and 51.5%) of N22 and PB-1121, respectively with respect to the control. When Spd application under HS was compared with HS alone, it was found that starch content was increased in both the genotypes in case of both flag leaf and spikelets. In case of spikelets higher increase in starch content was noticed in N22 (55%) as compared to PB-1121 (23%).

### Effect of Spd on total antioxidant capacity and enzymes activity

To underline the decrease in the accumulation of H_2_O_2_ and MDA contents, we also analyzed TAC and corresponding enzymes’ activities. TAC showed significant interaction in T of flag leaf (P<0.05), whereas G and T interation was presented in spikelets (P<0.001) of rice genotypes (**Table 3**). In flag leaf, maximum TAC was recorded in N22, while HS+Spd showed significant enhancement in TAC in PB-1121 (64.8 mMg^-1^FW). A similar trend was observed in spikelets of both the genotypes. A significant rise in TAC was recorded under HS+Spd treatment in both N22 (33.0 mM g^-1^FW) and PB-1121 (19.4 mM g^-1^FW).

**Figure 3:**
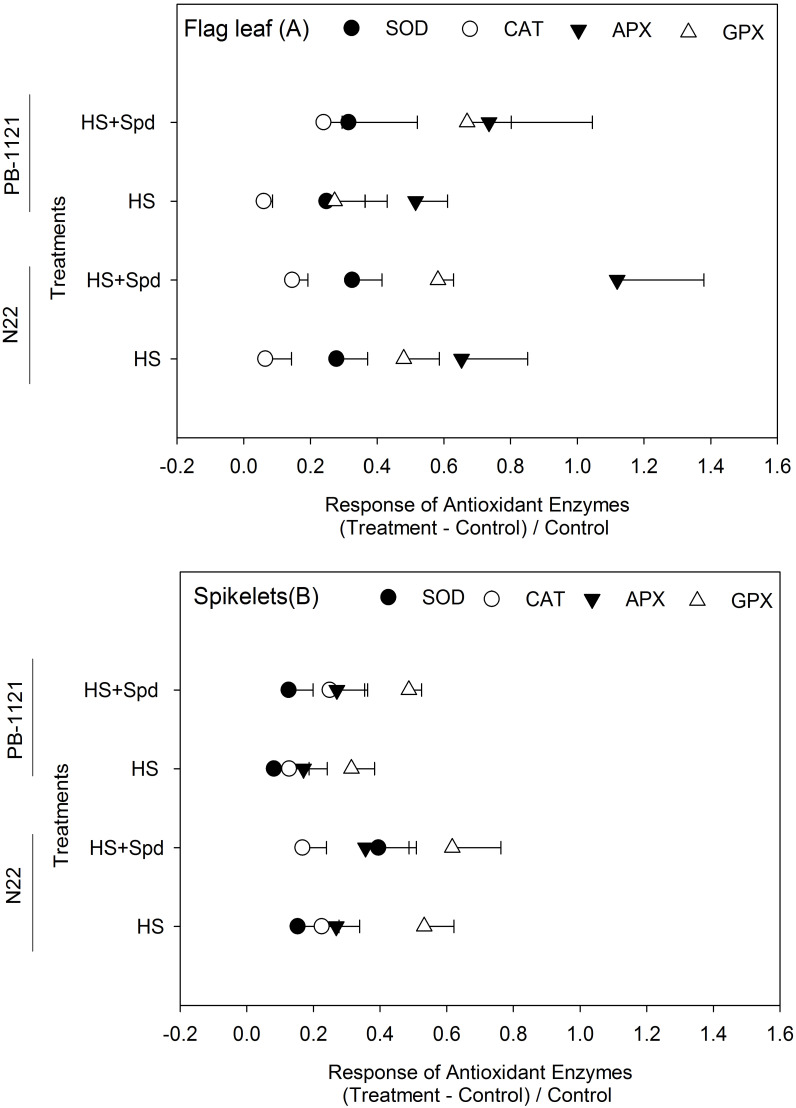
Response of antioxidant enzymes in contrasting rice genotypes (N22 and PB-1121) under different treatments in flag leaf **(A)** and spikelets **(B)**. Each point represents mean of five replicates. APX, Ascorbate peroxidase; CAT, Catalase; GPX, Guaiacol peroxidase; HS, Heat stress; Spd, Spermidine; SOD, Superoxide dismutase. SOD was expressed as units µmol; CAT and GPX was expressed as μmol of H_2_O_2_ consumption min−1 mg−1 protein and for APX, µmol ascorbate oxidized (APX) min^−1^ mg^−1^ protein, respectively. Least significant difference values at 5% for comparison are given in **Supplementary Table 2**.

Heat stress significantly induced activities of antioxidant enzymes *viz.* SOD, CAT, APX and GPX. SOD activity recorded significant variability in G and T in flag leaf (P<0.001-0.01), while in spikelet, significant G x T interactions were present (P<0.01) (**Figure 3**; **Supplementary Table 2**). In flag leaf, SOD activity was recorded in a range of 0.21-0.30 units mg^-1^protein. Under HS, its maximum activity was recorded in N22 (0.28 units mg^-1^protein) followed by PB-1121 (0.23 units mg^-1^protein). Spd application under HS significantly induced SOD activity in N22 (0.30 units mg^-1^protein) as well as PB-1121 (0.27 units mg^-1^protein). In spikelets, SOD activity was recorded in a range of 0.34-0.50 units mg^-1^protein. A similar trend was recorded in spikelets as well. CAT activity showed a significant difference among G and T in flag leaf (P<0.001-0.01) (**Figure 3**; **Supplementary Table 2**), whereas non-significant interaction was recorded in spikelets. Induction in CAT activity was recorded by Spd application in HS as shown in N22 (0.054 μmol H_2_O_2_ min^−1^g^−1^ protein) and PB-1121 (0.038 μmol H_2_O_2_ min^−1^g^−1^ protein) in flag leaf, whereas the marginal increase was recorded in spikelets of PB-1121.

APX activity showed a significant difference for G x T interaction among flag leaf (P<0.05), whereas in case of spikelets it was G and T interaction (P<0.001) (**Figure 3**; **Supplementary Table 2**). In flag leaf, APX activity was recorded in the range of 0.28-0.99 μmol ascorbate min^−1^g^−1^protein, whereas in spikelets, it ranged from 0.56 to 1.00 μmol ascorbate min^−1^g^−1^protein. Under HS, its maximum activity was induced in N22 (0.77 μmol ascorbate min^−1^g^−1^protein) followed by PB-1121 (0.42 μmol ascorbate min^−1^g^−1^protein). After Spd application under HS, significant APX activity was induced in N22 (0.99 μmol ascorbate min^−1^g^−1^protein) followed by PB-1121 (0.47 μmol ascorbate min^−1^g^−1^protein). A similar trend was recorded in spikelets where APX was recorded in the range of 0.56-1.0 μmol ascorbate min^−1^g^−1^protein. GPX activity was significant for G x T interaction in both flag leaf and spikelets (P<0.001) (**Figure 3**; **Supplementary Table 2**). In flag leaf, its activity ranged from 0.51–1.3 units, whereas in spikelet, the values ranged between 0.78-1.70 units. Higher GPX activity was recorded under HS+Spd in N22 (1.6 μmol H_2_O_2_ min^−1^g^−1^ protein). In general, Spd application significantly improved GPX activity in the flag leaf of PB-1121 among the genotypes and within treatments. In spikelets, its activity varied significantly in the range of 0.78–1.70 μmol H_2_O_2_ min^−1^g^−1^ protein with the highest activity in N22 (1.70 units).

### Effect of Spd on endogenous free-polyamines

**Figure 4:**
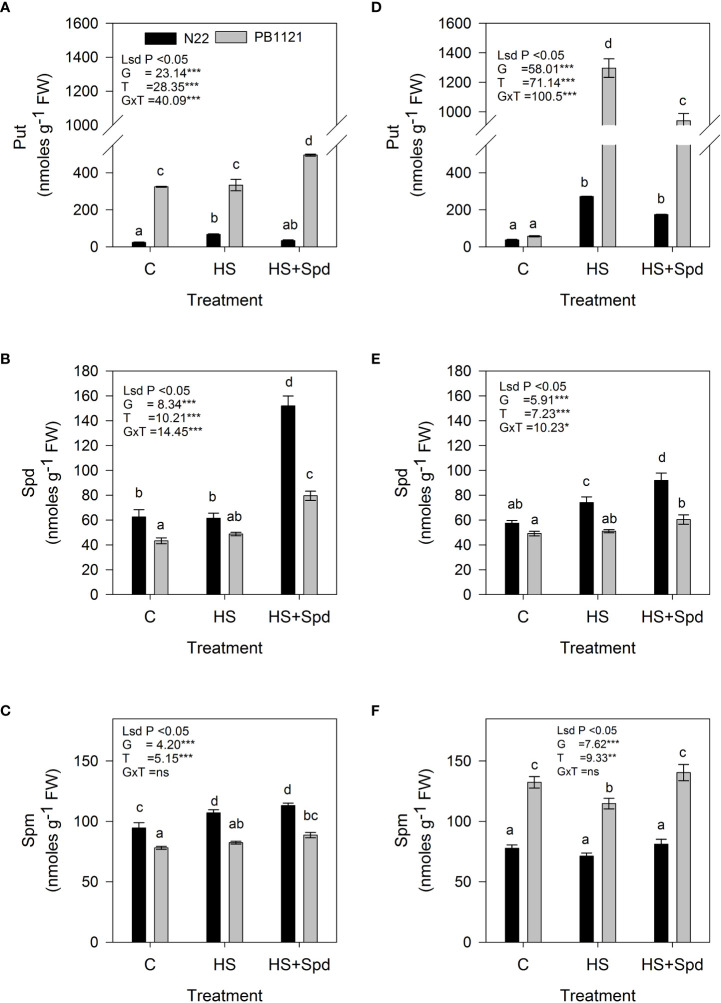
Effect of treatments on the endogenous content of polyamines in flag leaf **(A–C)** and spikelet **(D–F)** of rice genotypes. Bars indicate mean ± SE. Comparison of means was obtained from Tukey’s honestly significant difference test and means with the same letter are not significantly different at 5%. Fw, Fresh weight; Put, Putrescine; Spd, Spermidine; Spm, Spermine; LSD, least significant difference; G, Genotypes; T, treatment; GxT, Interaction between genotype and treatment. Significance level: *P < 0.05; **P < 0.01; ***P < 0.001; ns, nonsignificant.

Endogenous Put, Spd and Spm contents (units; nmoles g^-1^FW) showed significant variation among G and T in both flag leaf and spikelets (P<0.001-0.01) (**Figure 4**). Across the genotypes, endogenous Put levels in flag leaf varied significantly in the range of 23–495 nmoles g^-1^FW on the 7^th^ day of the treatment (**Figure 4A**). A significantly higher endogenous Put level under HS was recorded in Pusa-1121 (333 nmoles g^-1^FW), while it was comparatively lower in N22 (67 nmoles g^-1^FW). Compared to HS alone, Spd foliar application under HS significantly increased Put contents of PB-1121 by 48.6% while lowering its content in N22 by 50%. In spikelets significant increase in Put contents was recorded in both N22 and PB-1121 (271.8 and 1296 units, respectively) under HS. Spd application under HS significantly decreased Put contents in spikelets of both N22 and PB-1121 (35.9% and 27.0%, respectively) (**Figure 4D**).

Among the genotypes, endogenous Spd level in flag leaf varied significantly in the range of 43–151 nmoles g^-1^FW (**Figure 4B**) on the 7^th^ day of treatment. A significantly higher endogenous level of Spd under HS was recorded in N22 (61.4 nmoles g^1^FW), while it was lower in PB-1121 (48.7 nmoles g^-1^FW). When compared to HS alone, Spd foliar application under HS significantly increased Spd contents of PB-1121 by 63.1%, while in N22, it increased by 147.1% in flag leaf. A similar trend was observed in spikelets, where a significant increase in Spd content was recorded in N22 (74 unit), which was lower in PB-1121 (51 unit) under HS. Exogenous application of Spd under HS significantly increased endogenous Spd content in spikelets of both N22 and PB-1121 (24.1% and 18%, respectively) (**Figure 4E**). Among the genotypes, endogenous Spm level in flag leaf varied significantly in the range of 78–113 nmoles g^-1^FW (**Figure 4C**) on the 7^th^ day of treatment. A significantly higher endogenous level of Spm under HS was recorded in both N22 (107 nmoles g^-1^FW) and PB-1121 (82.4 nmoles g^-1^FW). When compared with HS alone, Spd foliar application in HS significantly increased Spm content of PB-1121 by 7.6%, while in case of N22, it showed a non-significant increase under HS. A similar trend was observed in spikelets, where a significant increase in Spm content was observed in PB-1121 (114.74 units) which was lower in N22 (77.8 units) under HS. Spd application under HS significantly increased Spm content in spikelets of both N22 and PB-1121 (4.37 and 22.4%, respectively) with respect HS (**Figure 4F**).

## Discussion

Heat stress (HS), particularly at the flowering stage, has detrimental effects on rice grain yield and quality (Barnabás, et al., 2008; Lyman et al., 2013; Jagadish et al., 2015; Jagadish, 2020). Predication suggested that short spells of heat spikes at the flowering stage have critical effects on rice (Krishnan et al., 2011). Therefore, increasing stress tolerance in rice at the most sensitive stage, i.e. flowering, is an ideal strategy to develop future climate-resilient varieties (Horie et al., 1996). Heat stress tolerance is majorly contributed by the robust antioxidant mechanism in crops like rice (Bahuguna et al., 2015), chickpea (Kumar et al., 2013) and many other crops (Hasanuzzaman et al., 2020). This mechanism has contributed towards high spikelet fertility and reproductive success under HS in rice (Bahuguna et al., 2015; Karwa et al., 2020).

It is documented that polyamines act as an essential regulatory component in response to various abiotic stresses in rice (Yang et al., 2007; Do et al., 2014; Karwa et al., 2020). Some reports of Spd for alleviating stress were presented under drought (Farooq et al., 2009) and heat (Mostofa et al., 2014) stresses. However, no studies are presented at the flowering stage. Therefore, this study was planned to address the vital gap, i.e. which polyamine regulates the defence mechanism under HS and how it helps maintain spikelet fertility and grain yield components under HS.

**Figure 5:**
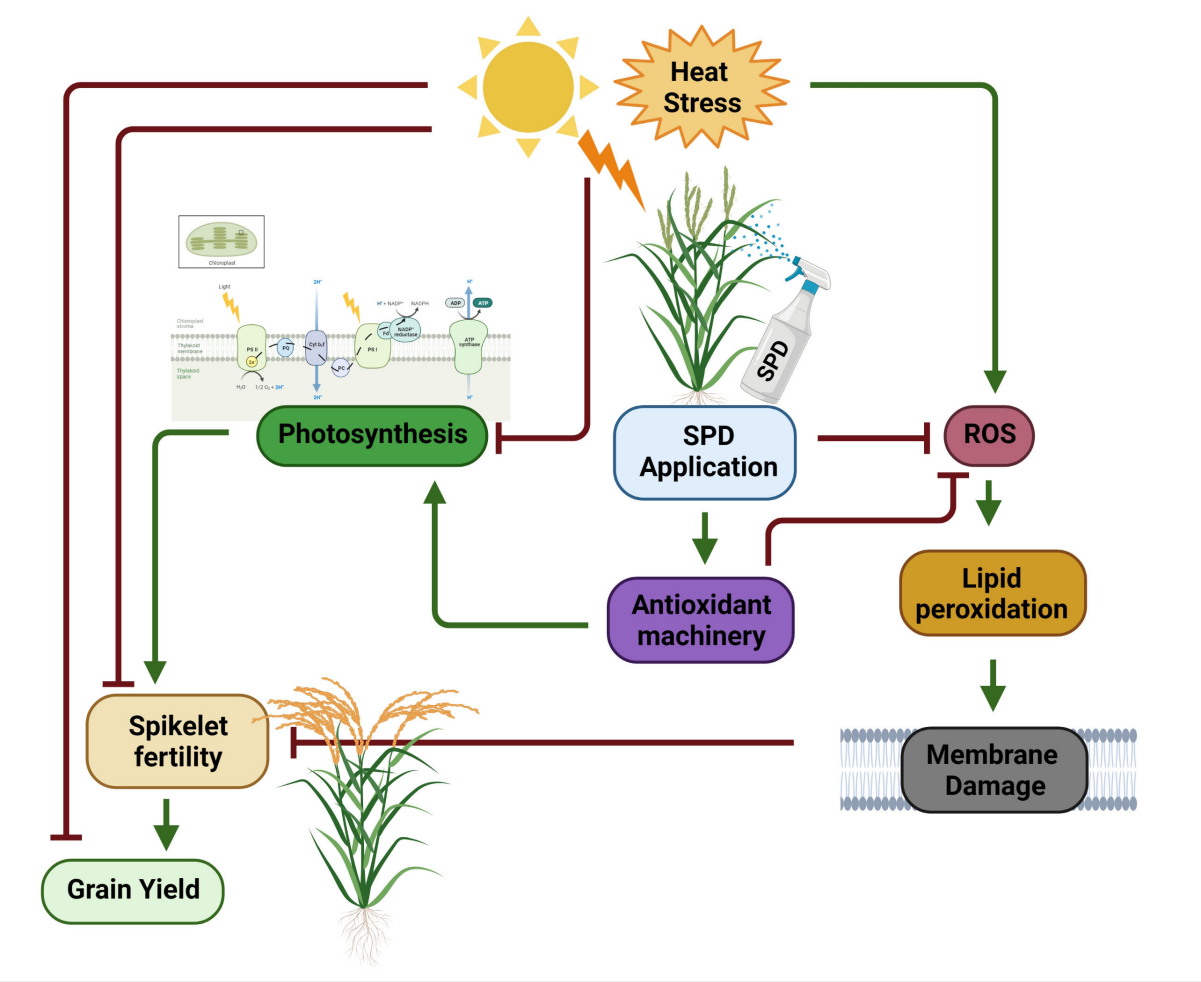
Exogenous Spermidine mediated response under heat stress in rice. Heat stress negatively imapcts photosynthesis, spikelet fertility and consequently grain yield in rice. Heat sress causes oxidative stress by producing ROS which leads to lipid peroxidation and thereby cell membrane damage. Damaged cell membrane often reduces spikelet fertility. Foliar application of Spd under heat stress induces antioxidant machinery (ROS scavenging mechanism) in rice plants which increases total antioxidant capacity resulting in higher photosynthesis, spikelet fertility and improved grain yield under HS. SPD, spermidine; ROS, Reactive oxygen species. This figure is created by Biorender.com.

Heat stress at the flowering stage showed adverse effects on spikelet fertility and grain yield in PB-1121. However, in the case of N22, lower reduction was recorded (**Table 2**), illustrating HS resilience in the latter genotype. Qi and Wu (2022) has suggested that spikelet fertility in rice plants under HS is primarily attributed to poor pollination manifesting. It has also been suggested that under HS, pollen viability and pollen dehiscence are severely affected, which would ultimately result in a decline in spikelet fertility when exposed during the flowering stage (Jagadish et al., 2010; Bahuguna et al., 2015; Karwa et al., 2020). Another reason was reported by Zhang et al. (2018) who explained that cross talk among auxins and ROS occur during HS, which inhibits pollen tube elongation in pistil. This decline in spikelet fertility results in grain yield reduction under HS. We recorded grain yield per plant reduction of 67% in PB-1121, while only 8% reduction was observed in N22 under HS. Spd is associated with pollen maturation and pollen tube growth in plants (Falasca et al., 2010; Aloisi et al., 2016). In our study, Spd application showed an alleviated effect of HS as its exogenous application improved spikelet fertility and grain yield per plant in both the genotypes (**Figure 5**).

Gaseous exchange traits *viz.* photosynthesis rate (i.e. CO_2_ uptake) and transpiration rate (i.e. H_2_O loss) are regulated through the stomatal behaviour of the plants. Stomatal pore opening and closing maintain the tissue temperature and movement of metabolites or signalling compounds in plants (Brownlee, 2001; Lake et al., 2001). Limitations in gas exchange can limit plant growth and development in various stresses (Wahid et al., 2007). In our study, it was observed that HS caused a higher decrease in *P_N_* in PB-1121 than N22 (**Figure 1A**). Degradation of chlorophyll and impartment of PSII system are the major factors for reduction in *P_N_* under HS (Efeuglee and Terzider, 2009). Therefore, these systems must have been severely impacted in case of PB-1121 as compared to N22 under HS. Polyamines regulate the voltage-dependent inward K^+^ channel in the plasma membrane of the guard cells and modulate stomatal aperture. All polyamines including Spd strongly induces closure of stomata under stress condition (Liu et al., 2000). Polyamine especially Spm and Spd induces secondary messenger in guard cell viz. NO causing stomatal closure. This increase in NO have a direct relation with H_2_O_2_ content (Gayatri et al., 2013). Recently, Spd application showed higher chlorophyll accumulation and relieved stomatal damage under HS that was maintaining P*_N_* under stress conditions (Yang et al., 2022). Our results are consistent with these findings as *P_N_* was directly linked with *gS* and E under HS, and a decrease in both the traits was recorded in PB-1121.

In contrast, higher E was recorded in N22, which maintained canopy temperature under HS (**Figures 1B, C**). The reduction in *gS* was due to the loss of leaf water potential under HS. We also annotated it in RWC under HS, which suggest that water content was reduced and membrane stability was also affected under HS (**Table 3**). Spd application under HS has improved water content, membrane stability, increased chlorophyll content and delayed leaf senescence in bentgrass (Li et al., 2015). In this study, we also observed similar trends with Spd application under HS where RWC and MSI were improved in PB-1121 followed by N22 with respect to HS. Torabian et al. (2018) have reported that exogenous Spd application alleviates water stress through protection of photosynthetic pigments, increase of proline and carotenoid contents and reduction of malondialdehyde content.

**Table 3:** Stress responses RWC, MSI, H_2_O_2_ accumulation, TBARS and TAC) in rice genotypes (N22 and PB-1121) under heat stress in flag leaf and spikelets.

Genotypes	Treatment	Spd applications	RWC (%)	MSI (%)	H_2_O_2_ (µmol g^-1^ FW)		TBARS (µmol g^-1^ FW)		TAC (mM g^-1^ FW)	
			Flag leaf	Flag leaf	Flag leaf	Spikelets	Flag leaf	Spikelets	Flag leaf	Spikelets
N22	Ambient	-Spd	88.3 ± 1.32	88.3 ± 1.24	11.5 ± 0.06	10.4 ± 0.01	20.4 ± 0.43	10.2 ± 0.56	56.5 ± 4.54	23.8 ± 3.39
	HS	-Spd	72.1 ± 0.85	74.1 ± 2.13	12.6 ± 0.14	10.5 ± 0.01	22.1 ± 1.18	11.0 ± 0.62	57.3 ± 1.79	23.1 ± 0.88
		+Spd	78.7 ± 1.03	77.7 ± 2.50	12.4 ± 0.04	10.5 ± 0.01	19.7 ± 0.54	10.9 ± 0.45	60.1 ± 1.83	33.0 ± 2.09
PB-1121	Ambient	-Spd	87.2 ± 0.91	81.9 ± 2.76	12.9 ± 0.19	10.5 ± 0.03	28.1 ± 2.05	31.0 ± 1.40	50.5 ± 2.08	13.6 ± 0.95
	HS	-Spd	61.8 ± 1.28	61.8 ± 1.58	15.2 ± 0.15	11.7 ± 0.02	40.6 ± 0.97	67.8 ± 2.29	53.3 ± 2.24	11.1 ± 0.25
		+Spd	73.1 ± 1.07	65.3 ± 1.45	13.7 ± 0.34	10.8 ± 0.12	35.2 ± 2.72	50.2 ± 2.83	64.8 ± 0.41	19.4 ± 0.84
Lsd<0.05		G	1.94***	3.60***	0.32***	0.09***	2.75***	2.91***	ns	3.10***
		T	2.37***	4.40***	0.39***	0.11***	3.37**	3.57***	5.38*	3.80***
		G X T	3.36**	ns	0.55**	0.16***	4.77*	5.50***	ns	ns

Each point represents the mean of five replicates with their SE. Least significant difference (Lsd) P<0.05, 0.01 and 0.001 were denoted by *, **, and *** respectively, and ns denotes non-significant; FW, Fresh weight; G, Genotypes; T, treatment; GxT, Interaction between genotype and treatment; HS, Heat stress; RWC, Relative water content; MSI, Membrane stability index; TBARS, lipid peroxidation; H_2_O_2_, reactive oxygen spices; TAC, Total antioxidant capacity; SE, Standard error; Spd, Spermidine.

Sugar and starch are the essential components for regulating plant metabolism under HS. The role of sugar in osmotic adjustment in case of rice and other crops has previously been discussed in many reports (Morsy et al., 2007). There are various studies with divergent points of view that sugar is either accumulated (Lu et al., 2009) or decreased (Liu et al., 2008; Liao et al., 2013) under HS. Our results suggest that under HS, total sugar and starch contents were reduced in flag leaf and spikelets. This can be explained by the fact that the source to sink relationship from flag leaf to spikelet is impaired under HS. Because of this, sugar transportation and its conversion to starch is also hindered (Kim et al., 2011; Ahmed et al., 2015). Present study suggest that, Spd application enhanced sugar accumulation and also supports the conversion of sugar into starch *via* regulating activity of soluble starch synthase, sucrose synthase and ADP glucose pyrophosphorylase (Wang et al., 2012; Fu et al., 2019). Fu et al. (2019) have reported that exogenous Spd application in rice upregulated the expression of starch synthetases genes which led to the increased accumulation of amylose in rice grains. This might be one of the reasons for significant variation of 1000 grain weight under HS with and without Spd application. Now, since N22 has more effective HS mitigation process/adaptive mechanism as compared to PB-1121, so in case of spikelets, these enzymes were more effectively operative under N22 as compared to PB-1121, when Spd was applied under HS, consequently resulting in higher increase in starch content in N22 as compared to PB-1121.

Like various other abiotic stresses, HS inhibits metabolic pathways, resulting in ROS production at the tissue level (Asada, 2006). ROS accumulation further induces membrane lipid damage in the form of increased MDA content. To counter ROS induced membrane damage, robust antioxidant machinery is required in plants (Gill and Tuteja, 2010). Conversely, the accumulation of ROS is also essential for stress signalling in plants; but beyond the optimal range, it damages metabolic processes in plants (Gupta et al., 2016). Our study indicates that H_2_O_2_ and MDA accumulated more in both flag leaf and spikelets of PB-1121 under HS than N22 (**Table 3**). These results are consistent with previous studies, which suggested that under HS, ROS and MDA accumulates in rice (Bahuguna et al., 2015; Karwa et al., 2020). Also, to metabolize these ROS radicals in cells, scavenging mechanisms in both enzymatic and non-enzymatic systems were reported in this crop (Basu et al., 2017; Szymańska et al., 2017). Various reports suggest that exogenous polyamines induce scavenging enzymes under stress conditions (Minocha et al., 2014, Liu et al., 2015); however, the nature of their interaction is a topic of debate (Groppa et al., 2001; Groppa et al., 2007). In our study, we recorded higher activities of APX and GPX under HS (**Figure 3**). Under HS, it was reported that downregulation of salicylic acid-binding protein (CAT) favours a rise in APX and GPX activities (Conrath et al., 1995; Dat et al., 1998). Upregulation of APX and GPX enzymes triggers a compensating mechanism to maintain H_2_O_2_ levels (Apel and Hirt, 2004; Sofo et al., 2015). In the present study, we observed that Spd application under HS significantly induced APX in flag leaf while in spikelets, GPX was induced under HS+Spd.

It was inferred that free polyamines (Put, Spd and Spm) levels behave genotype-dependently under various environmental conditions (Yang et al., 2007; Do et al., 2013; Pál et al., 2015). Endogenous polyamines serve as membrane surface stabilizers and increases the *P_N_* of plants by increasing photochemical efficiency of PSII (Shu et al., 2012). Further, it has been reported that exogenous application of polyamines might result in more substrate for proline biosynthesis specially under stress conditions (Farooq et al., 2009; Shi and Chan, 2014; Pál et al., 2018). Osmolytes like proline helps in osmotic adjustments, increases cell protoplasm concentration to maintain normal membrane function under heat and drought stresses. Exogenous Spd application has improved drought tolerance in bentgrass, maize and white clover by regulating endogenous polyamine metabolism (Li et al., 2015; Li et al., 2018; Li et al., 2016). Raziq et al. (2022) has found that exogenous Spd application has increased the biosynthesis of endogenous Spd and Spm from Put.

In the present study, HS increased Put content more in spikelets than flag leaf of PB-1121 (**Figures 4A, D**). These results are consistent with reports under heat (Mostafa et al., 2014) and drought stresses in rice (Mostafa et al., 2014). These results are collinear with the rise in endogenous Put accumulation, where a higher accumulation was observed in sensitive genotype—Pusa-1121. In this study, foliar application of Spd also alleviated Put content in spikelets and flag leaf of both the genotypes, where the most prominent rise was recorded in PB-1121. Put is biosynthesized from arginine *via* ADC or from ornithine *via* ODC. Qin et al. (2019) proposed that exogenous Spd augmented the content of Put as former increased *ADC1* and *ODC1* expression in case of *Malus domestica*.

Foliar application led to significant change in endogenous Spd content across the stress treatments in both the tissues, with the highest increase in N22 spikelets (**Figures 4B, E**). N22 consistently recorded higher Spd, while PB-1121 recorded lowest Spd across the treatments. Saha and Giri (2017) suggested that the increase in level of Spd was because of higher induction of *OsSPDS* in tolerant as compared to sensitive genotype. This can be the possible reason for rise in Spd content under HS. Exogenous Spd treatment has been reported to improve endogenous content together with imparting tolerance towards HS in rice (Mostafa et al., 2014) and towards drought stress in *Rosa damascena* (Hassan et al., 2018). We also observed a similar response in rice genotypes under HS.

It has been noted that another polyamine, Spm, was also elevated in the flag leaf of N22 and spikelets of PB-1121 under HS. Spd application resulted in increase in Spm endogenous content in PB-1121, whereas it was unchanged in N22. Elevated levels of both Spd and Spm has been reported to contribute towards plant stress tolerance under HS and other abiotic stresses (Liu et al., 2004; Do et al., 2014; Ikbal et al., 2014). For example, in case of oats, HS induced lipid peroxidation and membrane destabilization, but increased levels of Spd and Spm reversed this action by interaction with macromolecules (Tiburcio et al., 1994). In the present study, a marked decrease in Put accumulation was concomitant with an increase in endogenous levels of both Spd and Spm in N22. This accumulation pattern of the three polycations was previously reported in various crops under drought (Hassan et al., 2018) and salinity (Duan et al., 2008) stresses. Xu and Wu (2009) suggested that higher levels of Spd and Spm with respect to Put contributed towards water stress tolerance in pine. We found endogenous Put accumulation in the sensitive genotype, PB-1121. This might be due to lesser conversion of Put into Spd and Spm in the sensitive genotype. Bouchereau et al. (1999) proposed that a higher accumulation of Put indicates stress sensitivity of any organism. This is in agreement with the findings of Liu et al. (2004), where drought-tolerant wheat genotypes exhibited higher levels of Spd, whereas sensitive genotypes had more Put.

## Conclusion

The present study confirms that HS elevates oxidative stress in rice by increasing H_2_O_2_ and TBARS, decreasing photosynthesis, spikelet fertility and grain yield. Robust antioxidant machinery in tolerant genotype, N22 allowed minimum damage across the stress conditions. However, antioxidant defence in sensitive genotype was not at par to combat stress without applying Spd. Our study found that foliar application of Spd significantly induced ROS scavenging mechanism that resulted in increased photosynthesis, spikelet fertility, and grain yield under HS. This study highlights the probable association of Spd in HS tolerance in rice at the flowering stage *via* regulating robust antioxidant machinery, photosynthesis and spikelet fertility. There might be two possibilities for induction of antioxidant enzymes, either they bind with the polyamines and restore their functional stability and integrity, or catabolism of polyamine produces H_2_O_2_ byproduct, which acts as a signalling molecule and regulates series of signal transduction steps which activates antioxidant defence system to counter stress responses. Major research direction in the future should be to study the regulatory mechanism of Spd at molecular level and to study its role in stress signalling pathways. Development of overexpression lines or mutant lines of Spd synthase might help to elucidate the precise role of Spd to act as stress signal regulator or stress protecting compound under HS in rice. Also, future analyses focusing on manipulation of Spd metabolism and its environmental impact when applied exogenously are important to cope with the problem of climate change and for sustainable agricultural practices.

## Data availability statement

The original contributions presented in the study are included in the article/**Supplementary Material**. Further inquiries can be directed to the corresponding author.

## Author contributions

The authors have made the following declaration about their contributions. Conceived and designed the experiments: MP, SKU, SSA and SK. Performed the experiments: SK, JT, SM, AD and GKK. Analyzed and interpreted the data: SK and SKU. Performed statistical analysis: SK, SKU and AK. Drafted the manuscript: MP, SK, JT, SSA, SKU and VC. Edited and finalized the manuscript: MP, VC, JT, SKU and SK. All authors contributed to the article and approved the submitted version.

## Funding

Authors acknowledge the financial grant received from the Indian Council of Agricultural Research (ICAR), New Delhi, India, through National Innovations on Climate Resilient Agriculture (NICRA) Grant No.12-115 and ACRIP Project.

## Conflict of interest

The authors declare that they have no known competing financial interests or personal relationships that could have appeared to influence the work reported in this paper.

## Publisher’s note

All claims expressed in this article are solely those of the authors and do not necessarily represent those of their affiliated organizations, or those of the publisher, the editors and the reviewers. Any product that may be evaluated in this article, or claim that may be made by its manufacturer, is not guaranteed or endorsed by the publisher.

## Supplementary material

Supplementary Table 1 ANOVA for leaf gas exchange parameters of rice cultivars, N22 and PB-1121. Values presented are least significant difference (LSD) for each trait where *, **, *** indicate the significant difference and ns indicates non-significant difference; LSD with P< 0.05, 0.01 and 0.001 respectively. P_n_, Net photosynthetic rate; gS, stomatal conductance; E, transpiration rate; G, Genotype; T, Treatment; GxT, Interaction between genotype and treatment.

Supplementary Table 2 ANOVA for antioxidant enzymes of rice cultivars, N22 and PB-1121. Values presented are least significant difference (LSD) for each trait where *, **, *** indicate the significant difference and ns indicates non-significant difference; LSD with P < 0.05, 0.01 and 0.001 respectively. SOD, Superoxide dismutase; CAT, Catalase; APX, Ascorbate peroxidase; GPX, Guaiacol peroxidise; G, Genotype; T, Treatment; GxT, Interaction between genotype and treatment.
