# Supplementary material for: Spermidine exogenous application mollifies reproductive stage heat stress ramifications in rice
Source: Front Plant Sci. 2022 Dec 2;13:1027662. doi: 10.3389/fpls.2022.1027662 (PMC9755515; doi:10.3389/fpls.2022.1027662)
Supplement: Supplementary file 1 [file Presentation_1.pdf]

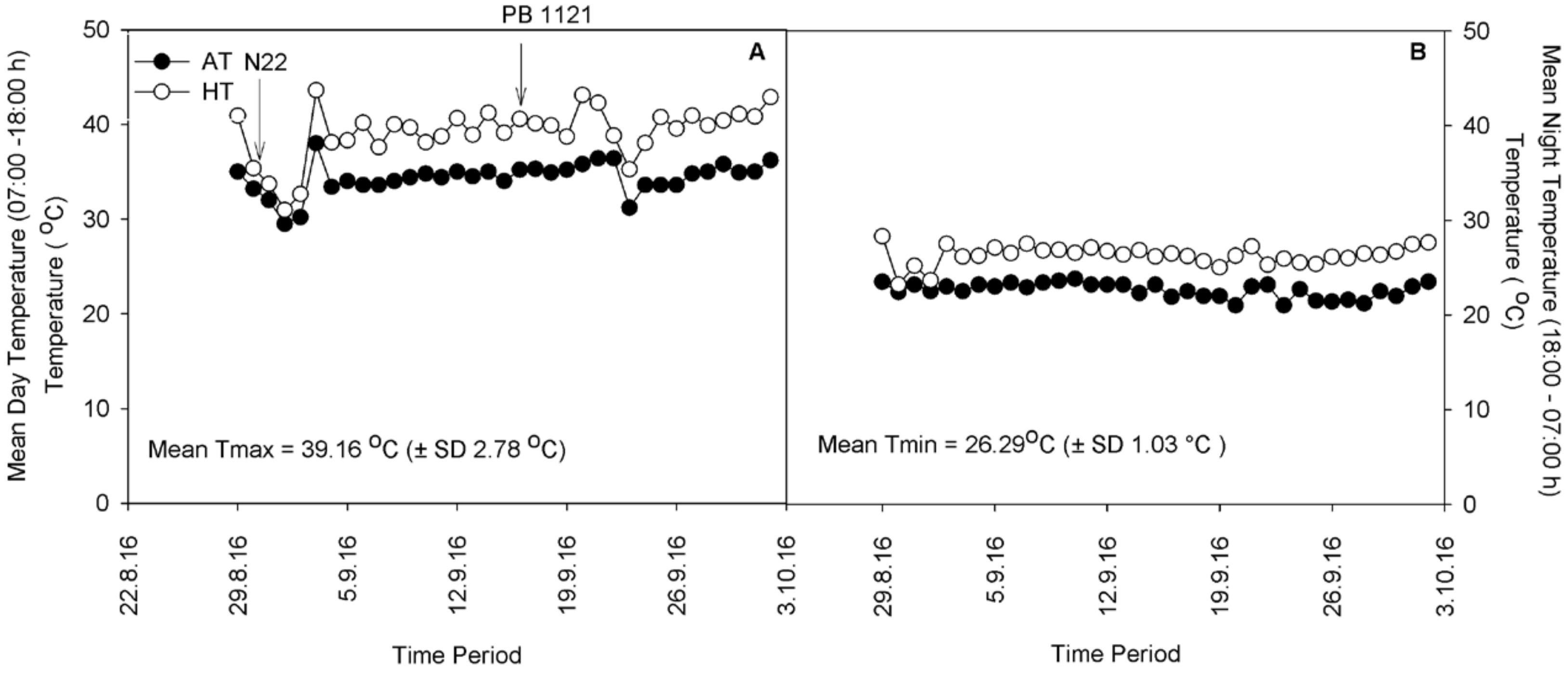

Supplementary Figure 1: Meteorological temperature data that prevailed during experiments. [A] showed daily mean day temperature, [B] showed mean night temperature during the experiment. Heat stress exposure during the experiment was started at heading and continued upto 100% flowering. Empty circles denote heat stress of the temperature tunnels and filled circle denotes ambient temperature of the field. Arrows denoted the day at which genotypes endowed for the high temperature at flowering stage. The overall temperature difference in tunnel temperature over control was 4.8ºC in the day and 3.7ºC at night.

Data File C:\CHEM32\1\DATA\SAURABH POLYAMINES NOV2017 2017-11-15 10-48-58\MIX 100PPM1.D
Sample Name: MIX 100PPM

Acq. Operator	: SUCHITRA	Seq. Line :	3
Acq. Instrument	: HPLC	Location :	Vial 19
Injection Date	: 11/15/2017 12:01:02 PM	Inj :	1
		Inj Volume :	20.0 µl
Acq. Method	: C:\CHEM32\1\DATA\SAURABH POLYAMINES NOV2017 2017-11-15 10-48-58\SAURABH POLYAMINES2017.M		
Last changed	: 11/15/2017 11:38:29 AM by SUCHITRA (modified after loading)		
Analysis Method	: C:\CHEM32\1\METHODS\NAVITA TOCOPHEROL.M		
Last changed	: 6/19/2018 4:01:34 PM by SUCHITRA		
Method Info	:		

Additional Info : Peak(s) manually integrated

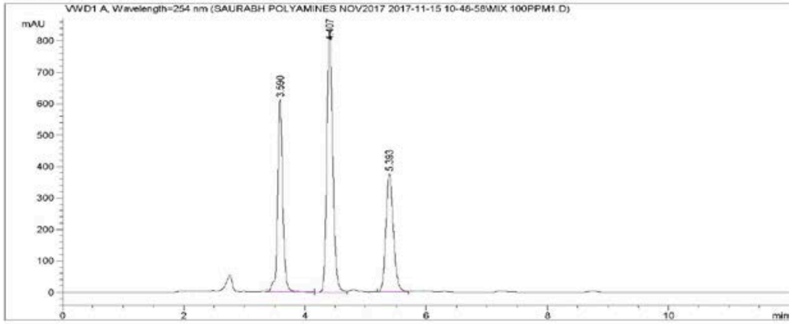

Area Percent Report

Sorted By : Signal
Multiplier: : 1.0000
Dilution: : 1.0000
Use Multiplier & Dilution Factor with ISTDs

Data File C:\CHEM32\1\DATA\SAURABH POLYAMINES NOV2017 2017-11-15 10-48-58\MIX 100PPM1.D
Sample Name: MIX 100PPM

Signal 1: VWD1 A, Wavelength=254 nm

Peak #	RetTime [min]	Type	Width [min]	Area mAU *s	Height [mAU]	Area %
1	3.590	VB	0.0887	3589.51099	612.62805	28.7821
2	4.407	BV	0.1045	5669.94189	829.12354	45.4638
3	5.393	VV	0.1334	3211.87231	375.22458	25.7541
Totals :				1.24713e4	1816.97617	

*** End of Report ***

Supplementary Figure 2: Chromatogram pattern and their respective retention time of three standard polyamines (Putrescine, Spermidine, and Spermine).
