# Supplementary material for: Spermidine exogenous application mollifies reproductive stage heat stress ramifications in rice
Source: Front Plant Sci. 2022 Dec 2;13:1027662. doi: 10.3389/fpls.2022.1027662 (PMC9755515; doi:10.3389/fpls.2022.1027662)
Supplement: Supplementary Table 1 — ANOVA for leaf gas exchange parameters of rice cultivars, N22 and PB-1121. Values presented are least significant difference (LSD) for each trait where *, **, *** indicate the significant difference and ns indicates non-significant difference; LSD with P< 0.05, 0.01 and 0.001 respectively. Pn, Net photosynthetic rate; gS, stomatal conductance; E, transpiration rate; G, Genotype; T, Treatment; GxT, Interaction between genotype and treatment. [file Table_1.docx]

Supplementary tables

Supplementary table 1: ANOVA for leaf gas exchange parameters of rice cultivars, N22 and PB-1121. Values presented are least significant difference (LSD) for each trait where*, **, *** indicate the significant difference and ns indicates non-significant difference; Least significant difference with P< 0.05, 0.01 and 0.001 respectively. P_n_= Net photosynthetic rate, gS= stomatal conductance, E= transpiration rate; G, Genotype; T, Treatment; GxT, Interaction between genotype and treatment.

| Traits | G | T | GxT |
| --- | --- | --- | --- |
| P_n_ | 0.73*** | 0.9*** | ns |
| gS | 0.049*** | 0.06*** | 0.087*** |
| E | 0..58*** | 0.71*** | 1.0* |

Supplementary table 2: ANOVA for antioxidant enzymes of rice cultivars, N22 and PB-1121. Values presented are least significant difference (LSD) for each trait where *, **, *** indicate the significant difference and ns indicates non-significant difference; Least significant difference with P< 0.05, 0.01 and 0.001 respectively. SOD, Superoxide dismutase; CAT, Catalase; APX, Ascorbate peroxidase; GPX, Guaiacol peroxidise; G, Genotype; T, Treatment; GxT, Interaction between genotype and treatment.

| Tissue | Traits | G | T | GxT |
| --- | --- | --- | --- | --- |
| Flag leaf | SOD | 0.02*** | 0.02** | ns |
|  | CAT | 0.004*** | 0.006** | ns |
|  | APX | 0.10*** | 0.12*** | 0.18* |
|  | GPX | 0.06*** | 0.07*** | 0.10*** |
|  |  |  |  |  |
|  |  |  |  |  |
|  |  | G | T | GxT |
| Spikelets | SOD | 0.02*** | 0.03*** | 0.04* |
|  | CAT | ns | 0.01* | ns |
|  | APX | 0.061*** | 0.086*** | ns |
|  | GPX | 0.027*** | 0.039*** | 0.04*** |
